# False-positive Serum Antiglomerular Basement Membrane Antibody due to Bovine Serum Albumin-containing Surgical Adhesive: A Case Report

**DOI:** 10.1016/j.xkme.2024.100880

**Published:** 2024-07-25

**Authors:** Ryuto Yoshida, Tatsuhiko Azegami, Shintaro Yamaguchi, Aika Hagiwara, Akihito Hishikawa, Norifumi Yoshimoto, Akinori Hashiguchi, Kaori Hayashi

**Affiliations:** 1 Division of Nephrology, Endocrinology and Metabolism, Department of Internal Medicine, Keio University School of Medicine, Tokyo, Japan; 2 Department of Pathology, Keio University School of Medicine, Tokyo, Japan

**Keywords:** Anti-glomerular basement membrane antibody, bovine serum albumin, false-positive, vancomycin

## Abstract

Antiglomerular basement membrane (GBM) disease has a poor prognosis. The rapid detection of serum anti-GBM antibody using an enzyme immunoassay, which has a high sensitivity and specificity, leads to an early diagnosis and improved prognosis. We report a case of acute kidney injury with false-positive anti-GBM antibody. A man in his early fifties underwent aortic arch replacement using bovine serum albumin (BSA)-containing surgical adhesion. After intravenous administration of vancomycin for a fever, he developed acute kidney injury without an abnormal urinalysis, and his anti-GBM antibody titer (fluorescence enzyme immunoassay [FEIA]) was 70.4 IU/mL. A kidney biopsy showed acute tubular injury and minor glomerular abnormalities without immunoglobulin G deposits, suggesting no evidence of anti-GBM glomerulonephritis. Consistent with the false-positive anti-GBM antibody test results, anti-GBM antibody determined using a chemiluminescent enzyme immunoassay was negative. A serum sample showed crossbinding to the FEIA plate from which the GBM antigen was removed. This finding indicated a nonspecific reaction to BSA, which contains a coating solution for the FEIA plate. This reaction was likely caused by anti-BSA antibody produced using BSA-containing surgical adhesion. Our findings suggest emerging challenges in diagnosing anti-GBM disease. Nephrologists must remain vigilant regarding false-positive anti-GBM antibody test results, particularly in cases evaluated with immunoassays that contain BSA.

## Introduction

Antiglomerular basement membrane (GBM) disease presents with necrotizing and crescentic glomerulonephritis caused by serum autoantibodies against the GBM, and this often leads to rapid progressive glomerulonephritis and pulmonary hemorrhage. Serum anti-GBM antibody, which is detected using an enzyme immunoassay (EIA), shows a high sensitivity and specificity in the diagnosis of anti-GBM disease.^1^ Anti-GBM disease has a poor prognosis for life and the kidney.^2^ Therefore, ensuring an accurate diagnosis and promptly initiating potent immunosuppressive therapies (eg, glucocorticoids, cyclophosphamide, and rituximab) and plasma exchange during the early stages of the clinical course are important. In this report, we present a patient with acute kidney injury who initially tested positive for anti-GBM antibody by fluorescence EIA (FEIA) but was later found to be false-positive for anti-GBM antibody. This case raises issues that can be faced in the clinical diagnosis of anti-GBM disease and highlights the importance of a comprehensive diagnostic approach to ensure appropriate and timely therapeutic intervention of this disease.

## Case Report

A man in his early 50s was admitted to our hospital for surgery to repair a thoracic aortic aneurysm. Aortic arch replacement was performed using BioGlue® (Artivion, Kennesaw, Georgia, USA), which is a surgical adhesive product containing bovine serum albumin (BSA) and glutaraldehyde. On the seventh day postoperation (7POD), he developed a fever and began shivering. Vancomycin and tazobactam/piperacillin were administered empirically. Subsequently, the serum creatinine concentration increased from 0.78 mg/dL at baseline to 3.2 mg/dL on 10POD. Laboratory data and urinary analysis on 15POD are shown in Table 1. On examination, the patient’s blood pressure was 105/65 mm Hg, heart rate was 71 beats/minute, and temperature was 36.9°C, without edema or a skin rash. A urinalysis showed protein (-), blood (-), trace leukocytes, and increased levels of biomarkers of kidney tubular injury. Despite the limited abnormal urinary findings, screening tests for nephritis were conducted because of rapid kidney function decline. Antinuclear antibody, myeloperoxidase anti-neutrophil cytoplasmic antibody (ANCA), and proteinase 3-ANCA test results were negative. The peak serum vancomycin concentration was 35.2 mg/dL on 11POD. Computed tomographic imaging showed no abnormalities in the kidneys, urinary tract, or lungs. Initially, vancomycin-associated acute tubular injury or tazobactam/piperacillin-associated acute interstitial nephritis were suspected as the cause of the acute kidney injury (AKI). However, the serum anti-GBM antibody determined using FEIA (EliA GBM Well®; Thermo Fisher Scientific, Waltham, Massachusetts, USA/Phadia AB, Uppsala, Sweden) was 70.4 IU/mL.

**Table 1:** Laboratory Data and Urinary Analysis on Post Operative Day 15

CBC	Result	Normal range	Chemistry	Result	Normal range	Urinary analysis	Result	Normal range
WBC	9,300 (/μL)	(3,300-8,600)	AST	12 (U/L)	(13-30)	pH	7.0	(5.0-7.5)
Neutro	78 (%)	(38.5-80.5)	ALT	12 (U/L)	(10-42)	Specific gravity	1.008	(1.002-1.030)
Lymph	5.0 (%)	(16.5-49.5)	LDH	240 (U/L)	(124-222)	Protein	(-)	
Mono	7.0 (%)	(2.0-10.0)	γ-GTP	109 (U/L)	(13-64)	Occult blood	(-)	
Eosino	8.0 (%)	(0.0-8.5)	TP	5.8 (g/dL)	(6.6-8.1)	Granular casts	(1+)	
RBC	2.75 (10^6/μL)	(4.35-5.55)	Alb	2.8 (g/dL)	(4.1-5.1)	RBC sediment	<1 (/HPF)	
Hct	27.0 (%)	(40.7-50.1)	Ca	8.7 (mg/dL)	(8.8-10.1)	WBC sediment	10-19 (/HPF)	
Hb	8.5 (g/dL)	(13.7-16.8)	SUN	42.2 (mg/dL)	(8-20)	β2-MG	10,662 (μg/L)	(0-200)
Plt	70.8 (10^4/μL)	(158-348)	Cre	6.96 (mg/dL)	(0.65-1.07)	NAG	16.0 (IU/L)	(0-11.5)
			Na	140.9 (mEq/L)	(138-145)	α1MG	23.3 (mg/L)	(0-8.3)
			K	5.1 (mEq/L)	(3.6-4.8)			
			CRP	7.74 (mg/dL)	(0-0.14)			
		(0.8-1.2)	IgG	950 (mg/dL)	(861-1,747)			
		(24-34)	IgA	140 (mg/dL)	(93-393)			
		(0-0.9)	IgM	58 (mg/dL)	(33-183)			
			IgG4	66 (mg/dL)	(11-121)			
			C3	162 (mg/dL)	(73-138)			
			C4	33 (mg/dL)	(11-31)			
			CysC	2.94 (mg/dL)	(0.63-0.95)			
			ANA	<40 (U/mL)	(<40)			
			PR3-ANCA	<1.0 (U/mL)	(<1.0)			
			MPO-ANCA	<1.0 (U/mL)	(<1.0)			
			Anti-GBM antibody	70.4 (U/mL)	(0.0-6.9)			
			VCM	25.2 (mcg/dL)				
			HCV antibody	(-)				
			HBs antibody	(-)				
			HIV antibody	(-)				

*Note:* Conversion factors for units: serum creatinine in mg/dL to μmol/L, ×88.4; urea nitrogen in mg/dL to mmol/L, ×0.357.

Abbreviations:α1MG, alpha-1 microglobulin; Alb, albumin; ALT, alanine aminotransferase; ANA, antinuclear antibody; AST, aspartate aminotransferase; ß2MG, beta-2 microglobulin; C3, complement component 3; C4, complement component 4; Ca, calcium; Cre, creatinine; CRP, C-reactive protein; CysC, cystatin C; GBM, glomerular basement membrane; γ-GTP, γ-glutamine transpeptidase; Hb, hemoglobin; HBs, hepatitis B surface; HCV, hepatitis C virus; HIV, human immunodeficiency virus; IgA, immunoglobulin A; IgE, immunoglobulin E; IgG, immunoglobulin G; IgM, immunoglobulin M; K, potassium; LDH, lactate dehydrogenase; Lymph, lymphocytes; Mono, monocytes; MPO-ANCA, myeloperoxidase anti-neutrophil cytoplasmic antibody; Na, sodium; NAG, N-acetyl-β-D-glucosaminidase; Neutro, neutrophils; Hct, hematocrit; Plt, platelets; PR3-ANCA, proteinase 3 anti-neutrophil cytoplasmic antibody; RBC, red blood cells; SUN, serum urea nitrogen; TP, total protein; VCM, vancomycin; WBC, white blood cells.

**Figure 1:**
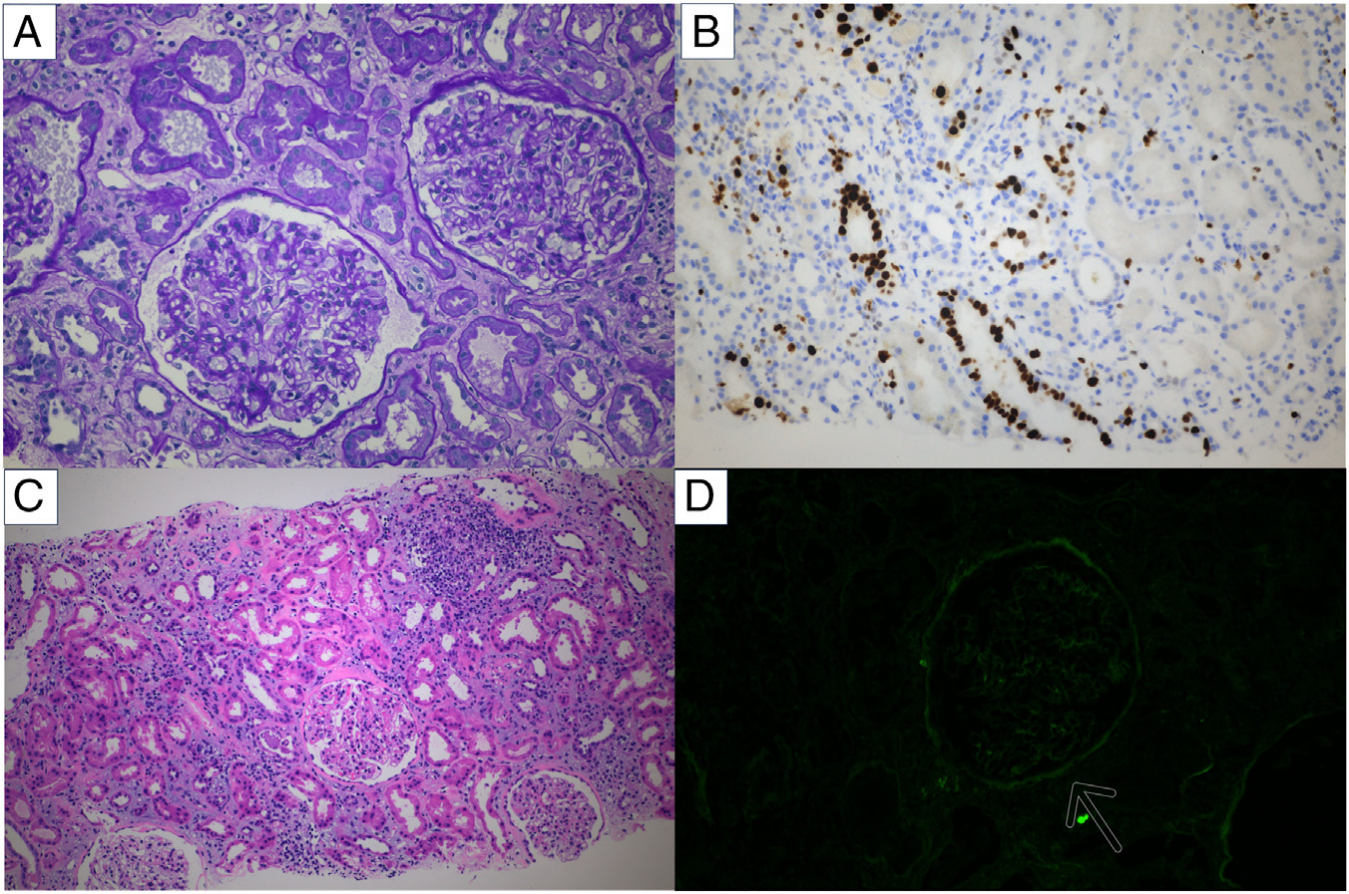
Renal biopsy findings. (A) Minor glomerular abnormality shown by periodic acid-Schiff staining. (B) Positive Ki-67 staining in the tubular epithelium. (C) Acute tubular injury with focal interstitial lymphocyte infiltration shown by hematoxylin and eosin staining. (D) Fluorescent antibody immunostaining (IgG (-)).

A kidney biopsy was performed to differentiate between anti-GBM disease and antibiotic-associated AKI on 16POD. At the same time, prednisolone was initiated at a dose of 30 mg/day for the possibility of acute interstitial nephritis. The histological findings of the kidney biopsy are shown in Figure 1. A light microscopy examination showed findings of tubular injury, such as flattening of epithelial cells with loss of the brush border and interstitial edema. Ki-67 staining showed a high positive rate in tubular cell nuclei, which suggested tubular regeneration. The interstitial inflammation was mild, and tubulitis was minimal. Nineteen glomeruli were obtained. However, the glomeruli showed no major change, and no crescents were identified. Vasculitis was not detected. An immunofluorescence examination showed negative staining for immunoglobulins and complements. The histological findings suggested that vancomycin-induced acute tubular injury was the most feasible underlying diagnosis. Therefore, we discontinued prednisolone 4 days after initiating treatment. Serum creatinine concentrations gradually improved and reached 0.84 mg/dL on 43POD. The anti-GBM antibody was remeasured using the chemiluminescent EIA method, but the test result was negative. We suspected that the initial positive test result of the serum anti-GBM antibody using the FEIA method was a false-positive test result. To confirm this suspicion, we retested the patient’s serum sample using an FEIA plate from which the GBM antigen had been removed, and the test result was positive. The positive test result was likely a nonspecific reaction to the coating solution, which contains BSA. Anti-BSA antibodies that can be produced owing to the use of BioGlue, which may have been the possible cause of the false-positive test result.

## Discussion

Although serum anti-GBM antibody shows a high sensitivity and specificity, as seen in this case, it can occasionally produce false-positive test results because of nonspecific reactions with the coating solution in an EIA plate. Therefore, nephrologists should use a comprehensive diagnostic approach for diagnosing anti-GBM disease by considering not only the serum anti-GBM antibody titer but also the patient’s clinical symptoms and a urinary analysis.

The serum anti-GBM antibody, known for its remarkable sensitivity and specificity, plays a pivotal role in diagnosing anti-GBM diseases. The sensitivity and specificity of this antibody may vary depending on the test assays used. A recent study compared various serum anti-GBM antibody assays with biopsy results from 103 serum samples.^1^ This previous study used the Elia GBM® assay, which was initially used in this case, and it showed a sensitivity of 94.7% and a specificity of 100.0%. A comprehensive meta-analysis that examined the accuracy of serum anti-GBM antibody tests across various assays reported a sensitivity of 93% (95% confidence interval: 84%-97%) and a specificity of 97% (95% confidence interval: 94%-99%).^3^ Therefore, because serum anti-GBM antibody testing consistently shows a high level of specificity, it is a useful tool for diagnosing anti-GBM diseases.

However, several reports have raised concerns when interpreting the results of a serum anti-GBM antibody (Table 2). Autoantibodies against the GBM are possibly induced owing to prior injury and inflammation of the basement membrane of the glomerulus and alveoli and are detected in serum. In several cases, these autoantibodies do not bind to the GBM and cause injury to the GBM. In a study involving cases of double positivity for ANCA and anti-GBM antibodies, kidney biopsy findings showed that 2 out of 25 (8%) cases showed pauci-immune glomeruli, suggesting that anti-GBM antibodies are not involved in kidney damage.^4^ Inflammation associated with ANCA-associated vasculitis, leading to damage in the GBM, represents a plausible mechanism in such instances of kidney injury. Moreover, the detection of serum anti-GBM antibody has been reported in human immunodeficiency virus (HIV)-infected patients without kidney involvement.^5^ This finding is likely because of the activation of humoral immunity, which can trigger the production of various autoantibodies, including anti-GBM antibodies. In our case, ANCA positivity and HIV infection that could potentially induce serum non-pathogenic anti-GBM antibodies were ruled out.

**Table 2:** Summary of Conditions that can Cause False-positive Serum Anti-GBM Antibody Results

Types of Causes	Examples
GBM injury	ANCA double positive
Activation of the humoral immunity	HIV
Nonspecific reactions (anti-BSA antibody)	Milk allergy Surgical adhesion (this case)

Abbreviations: ANCA, anti-neutrophil cytoplasmic antibody; BSA, bovine serum albumin; GBM, glomerular basement membrane; HIV, human immunodeficiency virus.

False-positive results because of nonspecific reactions to the test system have also been reported despite the absence of anti-GBM antibodies in the serum. Rüster et al^6^ reported a unique mechanism that can lead to false-positive anti-GBM antibody test results. These researchers documented a case in which a patient with a milk allergy developed antibodies against BSA, which is the blocking agent used in the coating solution of the anti-GBM antibody test kit. This immune response resulted in a nonspecific reaction and a false-positive test result. False-positive test results because of anti-BSA antibody have been reported in several fields. According to a previous report in patients with rheumatoid arthritis, the presence of anti-BSA antibody severely interferes with the anti-tumor necrosis factor antibody test results only in enzyme-linked immunoassays, which contain BSA as a blocking agent.^7^ Therefore, we further investigated the potential factors that could enhance the production of anti-BSA antibodies in our case. The patient had undergone surgery involving the use of BioGlue®, which is commonly used in cardiac surgery. Studies have shown that exposure to BSA-containing products following thoracic surgery can elevate anti-BSA antibody levels.^8^ We did not have an assay system to measure anti-BSA antibody at our facility, so we were unable to directly confirm its presence in this case. However, consequently, in our patient, exposure to the BSA-containing product likely led to the production of anti-BSA antibodies, subsequently resulting in false-positive serum anti-GBM antibody test results.

In conclusion, this case indicates the emerging challenges in diagnosing anti-GBM disease. Nephrologists must remain vigilant regarding the potential for false-positive serum anti-GBM antibody test results, particularly in cases evaluated with immunoassays containing BSA as the blocking agent and in cases involving the use of BSA-containing products.
